# Magnetic Nanoparticles as Effective Heavy Ion Adsorbers in Natural Samples

**DOI:** 10.3390/s22093297

**Published:** 2022-04-25

**Authors:** Urszula Klekotka, Ewelina Wińska, Elżbieta Zambrzycka-Szelewa, Dariusz Satuła, Beata Kalska-Szostko

**Affiliations:** 1 Faculty of Chemistry, University of Bialystok, Ciolkowskiego 1K, 15-245 Bialystok, Poland; u.klekotka@uwb.edu.pl (U.K.); ewelinawinska@wp.pl (E.W.); elazamb@uwb.edu.pl (E.Z.-S.); 2 Faculty of Physics, University of Bialystok, Ciolkowskiego 1L, 15-245 Bialystok, Poland; d.satula@uwb.edu.pl

**Keywords:** ferrite nanoparticles, heavy metal detection, food, pollution, particles functionalization

## Abstract

This paper refers to research based on tests completed on the adsorption of heavy metal ions (Pb^2+^, Cu^2+^, Cd^2+^) from selected natural liquid samples such as apple, tomato, and potato juices using surface-functionalized Mn ferrite nanoparticles (Mn_0.2_Fe_2.8_O_4_). To determine the most efficient adsorption conditions of these heavy metals, the nanoparticles’ surfaces were modified with five different ligands (phthalic anhydride, succinic anhydride, acetic anhydride, 3-phosphonopropionic acid, and 16-phosphonohexadecanoic acid). To evaluate the success of the adsorption process, the resultant liquid samples were examined for the amount of residuals using the flame atomic absorption spectroscopy method. The Mn ferrite particles selected for these tests were first characterized physicochemically by the following methods: transmission electron microscopy, scanning electron microscopy, X-ray diffraction, IR spectroscopy, Mössbauer spectroscopy.

## 1. Introduction

At present, environmental contamination from heavy metals is a highly urgent subject for scientists. Heavy metals are toxic to plants, animals, and humans [1]. Ubiquitous heavy metals cause a threat to human health and life. For this reason, it is important to effectively detect them and prevent poisoning [2]. Properly modified nanoparticles can capture many substances (ions or compounds) from various types of solutions (natural or artificial) and be successfully used as detectors or removal centers for these substances [3]. Especially effective in such instances are surface-functionalized magnetic nanoparticles which can be easily manipulated by external magnetic field [4].

Therefore, it is crucial to remove dangerous impurities from the human diet and environment [5,6] or, at minimum, have information about their contribution values. Heavy metals occur as contaminants in food because of their prevalence in the environment, resulting from human activities. People can be exposed to these metals, for example, through the consumption of contaminated food or water. Their accumulation in the body leads to harmful effects over time [7]. The major heavy metals present in food are lead, cadmium, and copper [8,9]. Both the International Agency for Research on Cancer and the National Toxicology Program have recognized cadmium as a classified Group 1 carcinogen [10]. Cd accumulates in the circulatory system, heart, kidneys, and lungs. Additionally, it is very toxic to bones [8]. In contrast, lead (Pb) damages the respiratory and immune systems. This metal is very toxic, especially for children, because it damages their nervous system. In children’s bodies, no organ system is immune to the effects of lead poisoning [8]. Poisoning with copper can cause nausea and central nervous system injury, as well as renal insufficiency [11].

The threat of heavy metals is a direct result of their movement through the trophic chain from soil–plant–animal–human, potentially resulting in their accumulation in the human body [12,13].

**Figure 1:**
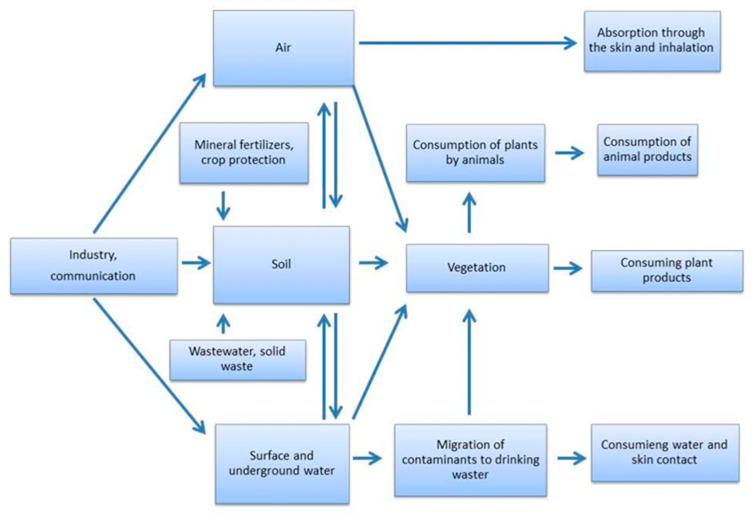
Schematic presentation of the possible transport of heavy metals in the environment.

The largest sources of heavy metals in soil comes from bedrock, industrial emissions, communications, and agriculture (Figure 1). The mining, metallurgy, and chemical industries are among the largest anthropogenic sources of soil pollution [12,13].

Magnetic nanoparticles can potentially be used to cleanse food of heavy metals due to their easy manipulation based on an external magnetic field. Moreover, a short contact time can ensure optimal conditions. In aqueous solutions, one of the important parameters is the pH of the contaminated mixture because of the formation of a thin layer of Fe-OH bonds on the surface of magnetite nanoparticles. This, in turn, can be protonated or deprotonated regardless of the pH. For easier removal of heavy metal ions, the surface of nanoparticles should be slightly negative, which can be obtained (most often reported) in solutions with a pH higher than six [14,15]. Nowadays, magnetic nanoparticles are used to treat water contaminated with heavy metals [14,16,17,18]. Suitable modification of the nanoparticles may result in a higher adsorption efficiency of heavy metals or an increase in the effects of the selected ion adsorption [19]. This can be achieved using magnesium–zinc ferrite, which successfully improves the removal of Cr(VI) and Ni(II) from solution [20], or calcium-doped ferrite which is most effective in the adsorption of Pd in comparison to other substances [21].

Summarizing the scattered data presented in the literature regarding the metal detectors based on nanoparticles, the most important are: the pH of the solution (its optimal value depends on the adsorbed ion) [22], the particles’ core composition (which is related to the size, shape, and surface morphology of the singular objects) [22,23], and the surfactant [24]. In this case, surfactants play roles not only as surface stabilizers, which prevent the aggregation of especially magnetic nanoparticles, but also in changing the surface characteristics to allow physical or chemical interactions. Additionally, surfactants separate the magnetic cores to a sufficient distance to prevent unfavorable magnetic attraction which reduces the effective surface area [25,26].

In this paper, we present research on the removal of selected heavy metal ions (Cd, Cu, Pb) from contaminated natural liquid samples (fruit and vegetables juices) by surface-modified (acetic anhydride, phthalic anhydride, succinic anhydride, 3-phosphonopropionic acid, and 16-phosphonohexadecanoic acid) Mn ferrite nanoparticles (Mn_0.2_Fe_2.8_O_4_). This study is a continuation of our previously obtained results and conclusions [27]. Therefore, similar experimental protocols were employed.

## 2. Materials and Methods

### 2.1. Reagents and Solutions

All chemicals used in this work were analytical grade and were used without any purification. FeCl_2_·4H_2_O, FeCl_3_·6H_2_O, tetrabutylammonium hydroxide (TBAOH) (40% in water), NH_3_ (25%), MnCl_2_ (anhydrous), CuSO_4_ (anhydrous), PbCl_2_ (anhydrous), Cd(NO_3_)_2_·4H_2_O, and acetic anhydride (AA C_4_H_6_O_3_) were purchased from Polish Chemical Reagents. Phthalic anhydride (PA C_8_H_4_O_3_), succinic anhydride (SA C_4_H_4_O_3_), 3-phosphonopropionic acid (3-PPA C_3_H_7_O_5_P), 16-phosphonohexadecanoic acid (16-PHDA C_16_H_33_O_5_P), and PBS (phosphate buffer sulfate) were received from Sigma–Aldrich. All chemicals were of ACS purity.

### 2.2. Apparatus

Nanoparticles used in the experiments were analyzed structurally, in terms of chemical composition, and magnetically by:

(i) X-ray diffractometry (XRD) (Agilent Technologies SuperNova diffractometer with a Mo micro-focused source (K_α2_ = 0.713067 Å))—placing a small amount of powder on a nylon loop using a high viscosity oil—to determine the crystal structure;
(ii) Transmission electron microscopy (TEM) (FEI Tecnai G2 X-TWIN 200 kV microscope—prefixing a drop of nanoparticle solution, on a carbon-covered 400 mesh Cu grid—to control particle morphology, shape, and size;
(iii) Infrared spectroscopy (IR) in the spectral range between 500 and 4000 cm^−1^ (using a Nicolet 6700 spectrometer working in transmission mode)—positioning a small amount of particle powder on a diamond window and squeezing via a stamp—to confirm surface functionalization;
(iv) Scanning electron microscope (INSPEC 60)—placing a small amount of particle powder on the microscopic table via conducting carbon tape—to examine the morphology of the obtained particle film;
(v) Mössbauer spectroscopy with a spectrometer working in constant acceleration mode with a ^57^Co in Rh matrix radioactive source—mixing the particle powder with BN and forming a disc—to establish the magnetic state of particles. The spectra were calibrated using α-Fe as a reference foil at room temperature (RT).

The amounts of Pb, Cu, and Cd elements in the tested solutions were measured using flame atomic absorption spectrometry (FAAS). Experiments were performed in a high-resolution continuum source atomic absorption spectrometer ContrAA 700 (Analytik Jena AG, Jena, Germany) equipped with a continuum light source—xenon short-arc lamp XBO 301 (GLE, Berlin, Germany) with the arc in a hot spot mode suitable for all elements’ determination. A double monochromator consisting of a prism pre-monochromator and a high-resolution echelle grating monochromator, along with a charge-coupled device (CCD) array detector with 588 pixels equipped with an air-acetylene flame was used for the determination of Pb, Cd, and Cu under optimized conditions of (a) Pb: burner height 7 mm, burner length 100 mm, air–C_2_H_2_ flow rate 75 L h^−1^; (b) Cu: burner height 4 mm, burner length 100 mm, air–C_2_H_2_ flow rate 65 L h^−1^; and (c) Cd: burner height 5 mm, burner length 100 mm, air–C_2_H_2_ flow rate 55 L h^−1^.

### 2.3. Synthesis of Mn^2+^ Doped Ferrite Nanoparticles

Magnetite nanoparticles doped with manganese were synthesized by co-precipitation of Fe(II), Mn(II), and Fe(III) chlorides in a 0.5% ammonia solution. As a surfactant, a water solution of TBAOH was used. In this case, about 20% of the iron (II) was replaced by Mn(II) [28,29]. The exact synthesis has been described in our previous papers [27]. The final sample was dried by rotary evaporation until a powder was obtained.

### 2.4. Modification of Nanoparticles PA, SA, AA, 3-PPA, and 16-PHDA

After synthesis, nanoparticles were modified with selected anhydrides (PA, SA, AA), and organophosphorus acids (3-PPA, 16-PHDA). Every step of the modification was conducted at room temperature. The attachment of anhydrides was conducted as follows: the respective anhydrous solutions were prepared in ethanol with a concentration of 0.14 M. Then, a solution of the corresponding anhydride was mixed with about 80 mg of nanoparticles (in powder form) and stirred for 4 h [24]. After this time, the solution was removed (with the assistance of an external magnetic field), and the powder was washed 3 times with ethanol and dried at RT (room temperature).

The modification with organophosphorus acids involved a different procedure. First, the nanoparticles were washed with acetone and ethanol. Then, 10 mg of nanoparticles (in powder form) was mixed with a 1mM solution of 3-PPA or 16-PHDA for 18 h. In the next step, a mixture of nanoparticles and organophosphorus acid solution was placed in an ultrasonic bath for 1 min, and then the solution was removed with the assistance of an external magnetic field. In the end, nanoparticles were washed 3 times with PBS solution and dried [30]. Modified nanoparticles were characterized using IR spectroscopy.

### 2.5. Preparation of Food Samples Solution for FAAS

In these studies, three types of vegetable/fruit juices were tested: tomato, apple, and potato, respectively. Squeezed juices from fresh fruits/vegetables were initially separated from the parenchyma with the use of a centrifuge, and the precipitate was separated from the solutions. The pH values of the respective solutions were: apple juice (2.07), tomato juice (4.98), potato juice (6.20). Then, the respective solutions were contaminated with each heavy metal ion at a concentration of 100 ppm. Then, the prepared juice samples were added to 2 mg of modified Mn-doped ferrite nanoparticles. The whole mixture of nanoparticles was stirred for 10 min. Afterward, the liquid was separated from the solid phase via the assistance of an external magnetic field. In the obtained solutions, the concentrations of Pb, Cu, and Cd ions were measured using the FAAS method.

## 3. Results

### 3.1. Physicochemical Characterization of Pristine and Modified Ferrite Nanoparticles

**Figure 2:**
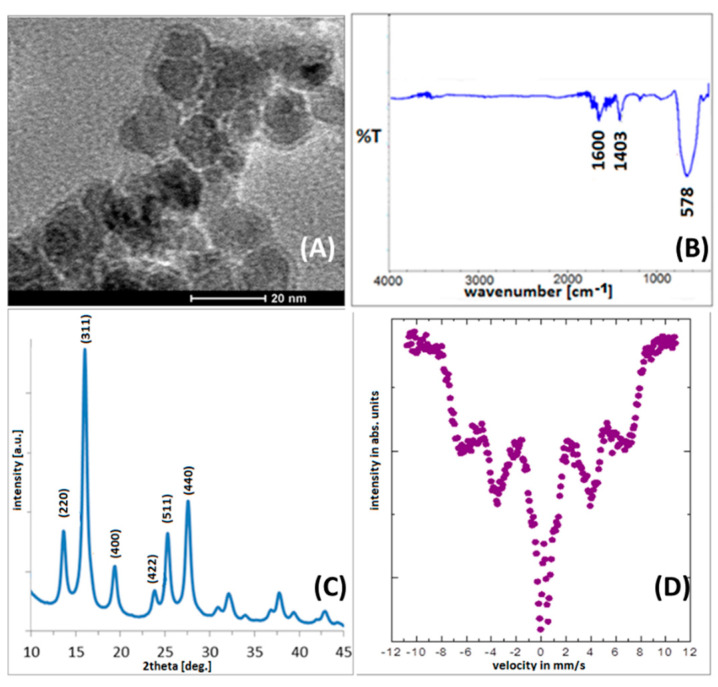
(**A**) TEM images of Mn_0.2_Fe_2.8_O_4_ nanoparticles; (**B**) IR spectra of pristine nanoparticles and with attached SA, AA, and 16-PHDA linkers, respectively; (**C**) X-ray diffractogram of Mn_0.2_Fe_2.8_O_4_; (**D**) Mössbauer spectrum of Mn_0.2_Fe_2.8_O_4_ nanoparticles.

The morphology of the fabricated pristine nanoparticles was characterized using TEM. As shown in Figure 2A, the obtained nanoparticles have round shapes and well-defined sizes with a narrow size distribution. The calculated nanoparticles’ diameter is about 15 ± 2 nm. Moreover, surfactant (TBAOH) shells can be also seen in the TEM image. Therefore, primary surface modification is confirmed [27].

The IR spectrum (Figure 2B) of Mn_0.2_Fe_2.8_O_4_ nanoparticles show only bands typical for the procedure used. The intensive signals present below 600 cm^−1^ originate from the Fe-O bonds in magnetite [31]. Bands around 1400–1600 cm^−1^ and below 3000 cm^−1^ are characteristic of O–H [32].

Depicted in Figure 2C, the X-ray diffractograms show a set of patterns that are typical for magnetite (or maghemite) structure without the reflections typical for other Mn or Fe oxide phases. These signals can be assigned Miller indexes of (220), (311), (400), (422), (511), and (440) [33]. The lattice constant calculated from the diffractograms (8.38 ± 0.02 Å) is consistent with the literature value of magnetite (8.39 ± 0.01 Å) [34]. The EDX measurements also showed that the percentage of Mn was 15%. This proves the substitution of Fe atoms by Mn^2+^ in the magnetite. Such a result confirms the successful incorporation of Mn into the primary structure [28].

The Mössbauer spectrum depicted in Figure 2D shows that Mn_0.2_Fe_2.8_O_4_ nanoparticles are in a different magnetic state than typical Fe_3_O_4_ at RT (room temperature) [27,35]. Such changes can be expected from dipole–dipole interactions between Mn^2+^ and Fe^2+^, Fe^3+^, when Mn^2+^ is incorporated into the structure [28]. At RT, Mn-doped particles are closer to a superparamagnetic blocking temperature in comparison to magnetite [28]. This fact weakens the interparticle interaction between separate nanoparticles and helps in their integration with third objects due to providing easier access to their surface [27].

### 3.2. Adsorption Tests

In this section, the results of the adsorption of heavy metals on the tested nanoparticles after the physicochemical characterization of the inorganic cores are presented. For this purpose, SEM images and IR spectra of the nanoparticles with proper surface functionalization after exposure to heavy metals are presented. Food samples contaminated with heavy metals before and after contact with the tested nanocomposites were analyzed by FAAS. The results of the percentage of value adsorbed are presented in Table 1.

### 3.3. Scanning Electron Microscopy

The morphology of the tested samples was imaged using SEM. The use of different juices caused significant changes in the particles’ film appearance resulting from the presence of variable organic matrixes that can be loosely adsorbed on the particles. Together with surfactants and juice constituents, the dried particles formed a relatively even film. However, in the solution, nanostructures were separated enough to have free and prolonged access to pollutants.

**Figure 3:**
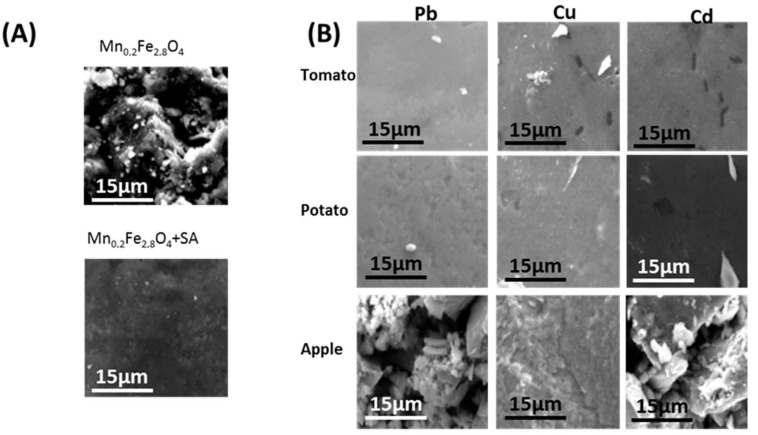
SEM images of films of (**A**) pristine Mn-doped ferrite nanoparticles; (**B**) nanoparticles after detection tests of heavy metals in selected food samples.

The film of pristine Mn_0.2_Fe_2.8_O_4_ particles (Figure 3A) was very rough because the surfactants used did not appear in large amounts as compared to the particles. Surface functionalization (Figure 3B) already causes the smoothing of the film because it increases the organic over the inorganic contribution. When the amount of surfactants and functional species dominates the system, particles can organize in a more relaxed manner because the interparticle magnetic interaction is weaker in this case. The bathing of particles in juice more strongly influenced the roughness/smoothing of the presented films. It was seen that the particles immersed in tomato and potato juices created a smooth film, while apple juice had the opposite effect regardless of the heavy metal tested. This was caused by the different compositions of the organic matrix of juices used.

### 3.4. Infrared Spectroscopy

**Figure 4:**
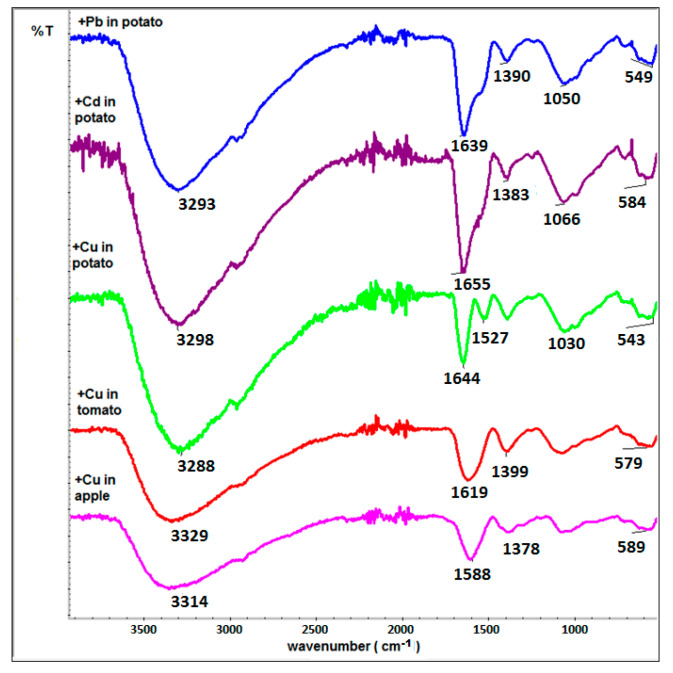
IR spectra of ferrite nanoparticles modified by SA after heavy metal detection from tested juices.

In Figure 2B, the IR spectrum of pristine Mn_0.2_Fe_2.8_O_4_ nanoparticles is presented. After surface modification and Pb, Cd, or Cu adsorption (selected spectra—Figure 4), spectra are richer. The more intensive bands at 1640 cm^−1^ originate from N-H bonds from primary amines, and bands around 1380 cm^−1^ which respond to C-H deformational vibrations are present [32]. Wide bonds around 3330 cm^−1^ are typical for O-H bonds in water, which is adsorbed on the nanoparticles’ surface since the organic matrix causes the presence of a very spongy surface coverage where water can be trapped. These bands are clearly related to the residues of the juice samples adsorbed on the surface of nanoparticles. As can be found in the literature, the modification of the IR spectra in the range 1300–1600 cm^−1^ can be related to the interaction of modified particles with heavy metals [24]. Therefore, the origin of signals in that range is most probably due to heavy ion adsorption.

### 3.5. Flame Atomic Absorption Spectroscopy

**Figure 5:**
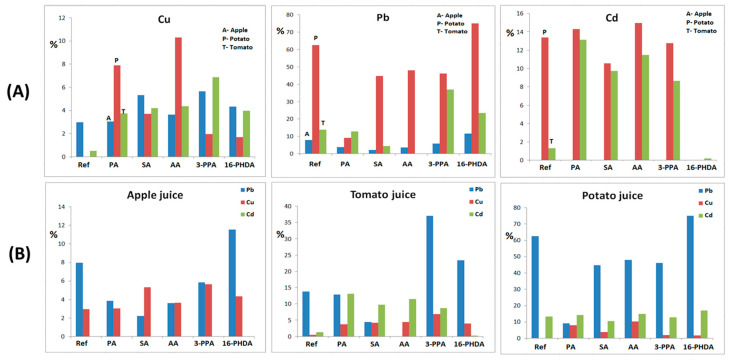
Graphical presentation of FAAS data: (**A**) juice dependence, (**B**) element dependence.

Juice solutions purposely contaminated with the respective elements were tested using the FAAS method. For this, each kind of solution was properly diluted and then expanded in flame. The adsorption data shows the following presence of detected ions in the samples (see Table 1 and Figure 5).

The data presented in Table 1 clearly indicate the juices in which certain heavy metal detection is the most effective. It is clear that Pb is much more efficiently adsorbed from potato juice in comparison to the other juices used (see Table 1 and Figure 5B). This is clearly connected with the pH value of the potato juice (6.02). The effect of pH on the adsorption of Pb^2+^ has been studied elsewhere, and it was estimated to be around 6–8 [24]. This is also in alignment with our previous studies, where the detection of heavy metals was tested in model water solutions [27]. Moreover, in this case, the most promising surface functionalization is connected with the presence of SA, 3-PPA, and 16-PHDA (due to its universality). The potato pure organic matrix also allows for the detection of a high number of Pb ions by pristine Mn_0.2_Fe_2.8_O_4_ NPs (almost 63%) and that modified by AA or SA (besides the most effective 16-PHDA). These results also confirm that the most universal functionalization for Pb detection was obtained by 16-PHDA. The situation is different in the case of Cu. Here, adsorption is much lower and the best linker cannot be clearly determined because its efficiency is not much different from the one used. Additionally, the pH effect does not play an equally important role for Pb^2+^. The general conclusion that can be made is that nanoparticles’ surfaces have to be functionalized, otherwise adsorption will not take place at all. For Cu in each juice (and pH), a different linker is the most active. The case of Cd adsorption is also very clear then; Cd^2+^ ions cannot be detected at all for a low pH, as is the case for apple juice (around 2) [36]. Similarly to Pb^2+^, the highest values were obtained in potato juice at pH 6, regardless of surface functionalization. The adsorption of Cd^2+^ is similar in the case of potato and tomato juices except for unmodified particles and 16-PHDA. The results show that in potato juice, Cd is detected regardless of the modifier used with almost equal efficiency. In this matrix, pristine NPs are also effective, but are very ineffective in tomato juice.

All these results suggest that many parameters must be taken into account when proper detectors are planned. All this is important in the interpretation of the adsorption effect. On the contrary, in the model water-based solutions, clear conclusions were obtained [24]. Therefore, a selective but not universal linker has been described. For future studies, the detection of a universal linker is required. Moreover, more studies on the pH effect are needed.

Figure 5 shows a graphical presentation of the FAAS results with respect to the identified element (A) or juice (B).

In Figure 5B, it is clear that pH strongly governs the adsorption capability of the presented elements more so than linkers. In apple juice, Cu adsorption is equally effective regardless of the linkers used. In contrast, for Pb, it evidently works much better as ‘naked’ particles or coated with 16-PHDA. The least effective is SA. In this environment, Cd is not detectable at all. In potato juice, Pb adsorption is very high, with some Cd adsorption as well. Cu is detected only on modified particles. Tomato juice is the most complex, whereby the adsorption of elements very strongly depends on the linkers present on the particles’ surface.

## 4. Conclusions

Detailed qualitative and quantitative studies show that detection from real matrixes (contaminated fruit extracts) is not an easy task. It is clear that that the analyzed elements, linkers used, organic matrix composition, and pH for detection are all important factors. All this leads to the conclusion that more studies on this subject are necessary, where the step-by-step process of the mentioned parameters are examined. In the studied series, the effective detection of Cd in potato and tomato juices by PA, SA, AA, and 3-PPA was evident. A high efficiency of AA in potato juice for each studied element was seen as well. The selectivity of adsorption related to the extracts and tested elements was also observed.

## Figures and Tables

**Table 1:** Percentage identification of elements in respective juices (columns) and selected modifiers (rows) (LOD—detection limit [36]).

Sample Type	% Adsorbed ± 0.05		
	Apple	Potato	Tomato
	I	II	III
**Pb**			
Mn_0.2_Fe_2.8_O_4_ NP’s	7.98	62.53	13.80
Mn_0.2_Fe_2.8_O_4_ + PA	3.84	9.19	12.82
Mn_0.2_Fe_2.8_O_4_ + SA	2.22	44.80	4.38
Mn_0.2_Fe_2.8_O_4_ + AA	3.62	48.00	<LOD
Mn_0.2_Fe_2.8_O_4_ + 3-PPA	5.83	46.22	37.01
Mn_0.2_Fe_2.8_O_4_ + 16-PHDA	11.52	75.02	23.38
**Cu**			
Mn_0.2_Fe_2.8_O_4_ NP’s	2.98	<LOD	0.50
Mn_0.2_Fe_2.8_O_4_ + PA	3.04	7.89	3.76
Mn_0.2_Fe_2.8_O_4_ + SA	5.32	3.72	4.21
Mn_0.2_Fe_2.8_O_4_ + AA	3.64	10.31	4.38
Mn_0.2_Fe_2.8_O_4_ + 3-PPA	5.66	1.97	6.89
Mn_0.2_Fe_2.8_O_4_ + 16-PHDA	4.33	1.69	3.98
**Cd**			
Mn_0.2_Fe_2.8_O_4_ NP’s	0.01	13.38	1.33
Mn_0.2_Fe_2.8_O_4_ + PA	0.02	14.3	13.13
Mn_0.2_Fe_2.8_O_4_ + SA	0.02	10.56	9.75
Mn_0.2_Fe_2.8_O_4_ + AA	0.03	14.93	11.47
Mn_0.2_Fe_2.8_O_4_ + 3-PPA	0.01	12.75	8.64
Mn_0.2_Fe_2.8_O_4_ + 16-PHDA	<LOD	16.89	0.21

## Data Availability

The raw/processed data required to reproduce these findings cannot be shared at this time as the data also form part of an ongoing study.
